# Communicating imaging findings in peritoneal mesothelioma: the impact of ‘PAUSE’ on surgical decision-making

**DOI:** 10.1186/s13244-021-01118-y

**Published:** 2021-11-24

**Authors:** Anuradha Chandramohan, Nehal Shah, Andrew Thrower, Norman John Carr, Rohin Mittal, Faheez Mohamed, Brendan Moran

**Affiliations:** 1 Christian Medical College, Vellore, 632004 India; 2 Department of Radiology, Basingstoke Hospital, Aldermaston Road, Basingstoke, RG24 9NA UK; 3 Peritoneal Malignancy Institute, Basingstoke Hospital, Aldermaston Road, Basingstoke, RG24 9NA UK

**Keywords:** Peritoneal mesothelioma, Complete cytoreduction, Radiological peritoneal cancer index, PAUSE, Imaging

## Abstract

The peritoneal cavity is the second commonest site of mesothelioma after the pleural cavity. There are five histological types of peritoneal mesothelioma with variable symptomatology, clinical presentation and prognosis. Cystic mesothelioma is a borderline malignant neoplasm with a favourable prognosis, well-differentiated papillary mesothelioma is generally a low-grade malignancy, and all other varieties such as epithelioid, sarcomatoid and biphasic mesothelioma are highly malignant types of peritoneal mesothelioma with poor prognosis. Malignant peritoneal mesothelioma was considered inevitably fatal prior to the introduction of cytoreductive surgery (CRS) and hyperthermic intraperitoneal chemotherapy (HIPEC) in selected cases where long-term survival and cure could be achieved. However, the survival benefits following CRS and HIPEC mainly depend on completeness of cytoreduction, which come at the cost of high morbidity and potential mortality. Using the acronym ‘PAUSE’, we aimed at describing the key imaging findings that impact surgical decision-making in patients with peritoneal mesothelioma. PAUSE stands for peritoneal cancer index, ascites and abdominal wall disease, unfavourable sites of involvement, small bowel and mesenteric disease and extraperitoneal disease. Reporting components of ‘PAUSE’ is crucial for patient selection. Despite limitations of CT in accurately depicting the volume of disease, describing findings in terms of PAUSE plays an important role in excluding patients who might not benefit from CRS and HIPEC.

## Key Points

- Imaging findings that impacts surgical decision making in peritoneal mesothelioma can be communicated effectively using the acronym PAUSE, which stands for Peritoneal Cancer Index, Ascites and abdominal wall disease, Unfavourable sites, Small bowel and mesenteric disease and Extraperitoneal disease.
- CT is the initial modality of choice for evaluating patients with peritoneal mesothelioma.
- CT is a good technique for identifying patients with high PCI disease, who may not benefit from CRS and HIPEC.

## Introduction

Peritoneal mesothelioma is a rare condition arising from the mesothelial cells lining the peritoneal cavity. The estimated incidence in men ranges from 0.5 to 3 per million/year and in women ranges from 0.2 to 2 per million/year. The peritoneal cavity is the second commonest site of mesothelioma after the pleura and it is estimated that 10–30% of all cases of mesothelioma occur in the peritoneal cavity [1, 2]. While there is a strong association between asbestos exposure and pleural mesothelioma, with 90% of sufferers having a history, this is only true for around half of those with peritoneal mesothelioma [3–6]. In addition, it seems that asbestos plays a smaller role in the development of peritoneal mesothelioma in women, only 23% of whom have a history of exposure [7]. Peritoneal mesothelioma also has reported associations with Mediterranean fever; exposure to erionite (a silicate fibre); radiation from thorotrast; papovavirus, simian virus 40; chronic peritonitis and BRCA gene mutations [1, 3, 8, 9].

## Clinical presentation

Peritoneal mesothelioma commonly presents with non-specific symptoms such as abdominal pain and distension [10, 11]. Patients with aggressive subtypes and those with advanced disease may present with rapidly progressing abdominal distension due to large volume ascites, omental disease or intestinal obstruction.

## Diagnosis

Incidental diagnosis of peritoneal mesothelioma during cross-sectional imaging for other conditions or at laparoscopy, or laparotomy, is not uncommon [10, 12]. Confirmation of the diagnosis is based on histological analysis of biopsies from the peritoneal cavity, obtained either by percutaneous image-guided biopsy, laparoscopy or laparotomy. Cytological analysis of ascitic fluid is of little diagnostic use in most cases [13]. Both the morphological analysis and the immunohistochemistry are crucial for the diagnosis of peritoneal mesothelioma. Immunohistochemical staining is positive for vimentin, calretinin, cytokeratin 5/6 (CK 5/6) and Wilm’s tumour-1 (WT-1), suggesting mesothelial origin, and is typically negative for thyroid transcription factor 1 (TTF 1), BErEP4 antibody, endothelial markers like CD31 and CD34, factor VIII and vascular endothelial growth factor receptor 3 [13]. Loss of expression of BAP1 (BRCA-associated protein 1) has been shown to have high specificity for differentiating peritoneal mesothelioma from benign mesothelial proliferation [14]. Tumour markers such as CA-125, CA15-3, mesothelin and osteopontin may be elevated in patients with peritoneal mesothelioma [15–17]. However, these markers are not useful for diagnosis, but may have a role in monitoring for progression or detection of recurrence after surgery.

## Histological types and their imaging appearance

There are five main histological types of peritoneal mesothelioma with widely varying clinical behaviour. Cystic mesothelioma is a borderline malignant neoplasm; well-differentiated papillary mesothelioma is a low-grade malignant subtype; and the other types such as epithelioid, sarcomatoid and biphasic subtypes of peritoneal mesothelioma are highly malignant. Desmoplastic, lymphohistiocytoid and deciduoid subtypes of peritoneal mesothelioma are other rare malignant mesothelioma subtypes.

## Cystic mesothelioma

Cystic mesothelioma is a rare neoplasm, estimated to account for 3–5% of peritoneal mesotheliomas and is most commonly found in young and middle-aged women [18, 19]. This entity has many synonyms such as peritoneal inclusion cysts, multicystic peritoneal mesothelioma and benign multicystic mesothelioma. It is increasingly recognised that cystic mesothelioma is a ‘borderline malignancy’ which recurs. The exact pathogenesis of cystic mesothelioma is unknown. Some consider it to be secondary to chronic peritoneal irritation caused by endometriosis, chronic pelvic inflammation or previous surgery [20]. Patients may present with non-specific abdominal pain and tenderness, deep pelvic pain, dyspareunia and urinary symptoms, but in many, the disease is found incidentally and the patient may be totally asymptomatic.

**Fig. 1:**
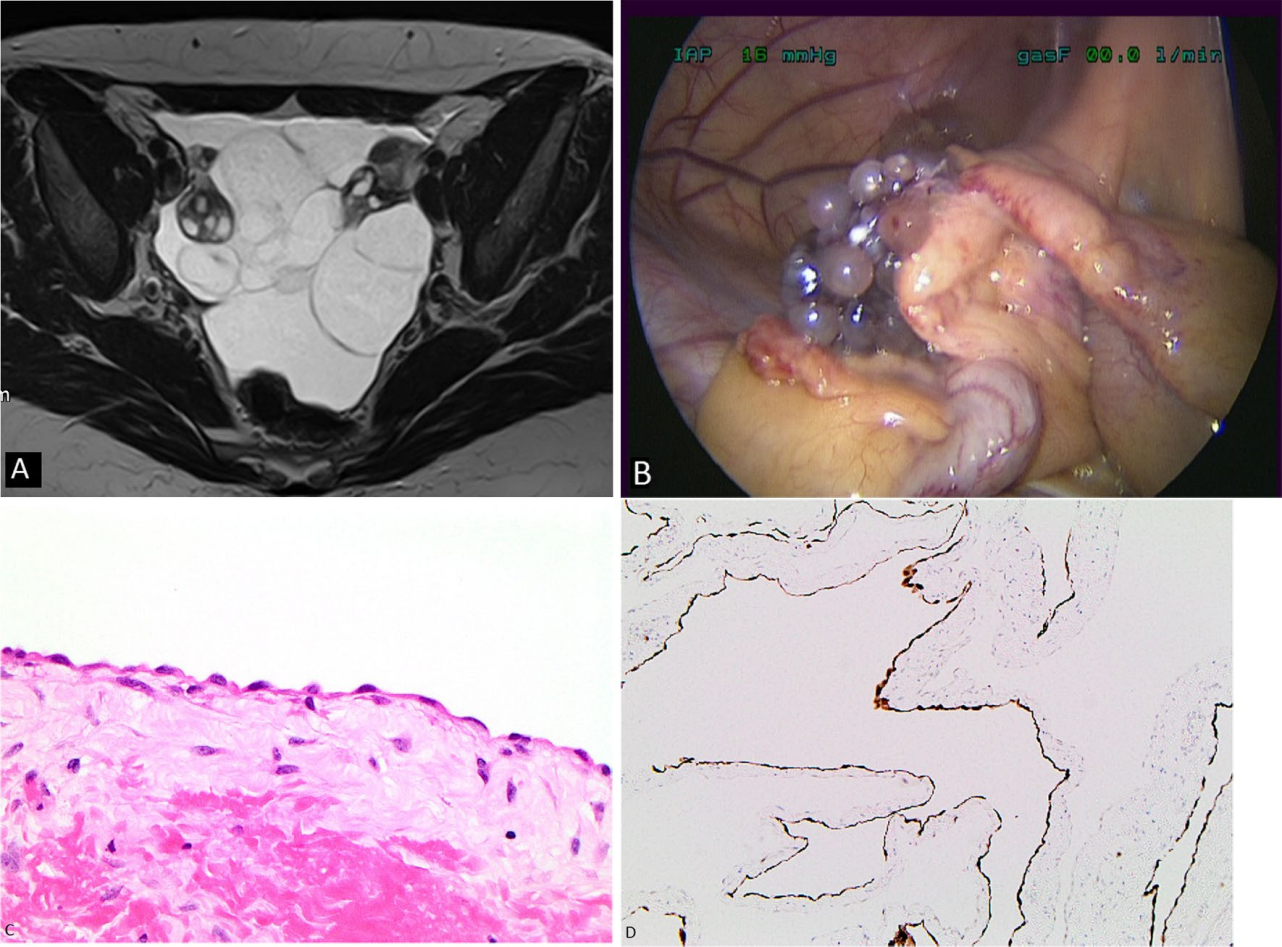
**a**–**d** 40-year-old female with cystic mesothelioma. **a** MRI T2 weighted axial image of the pelvis shows fluid intensity loculated cystic lesions surrounding the ovaries. **b** Intraoperative photograph showing the same findings. **c** High-power photomicrograph (H&E × 40) shows a flat layer of mesothelial cells lying on a loose fibrous stroma. **d** Low power photomicrograph (× 10) with calretinin immunohistochemical staining shows calretinin expression by the neoplastic cells which outline the shape of the cysts

**Fig. 2:**
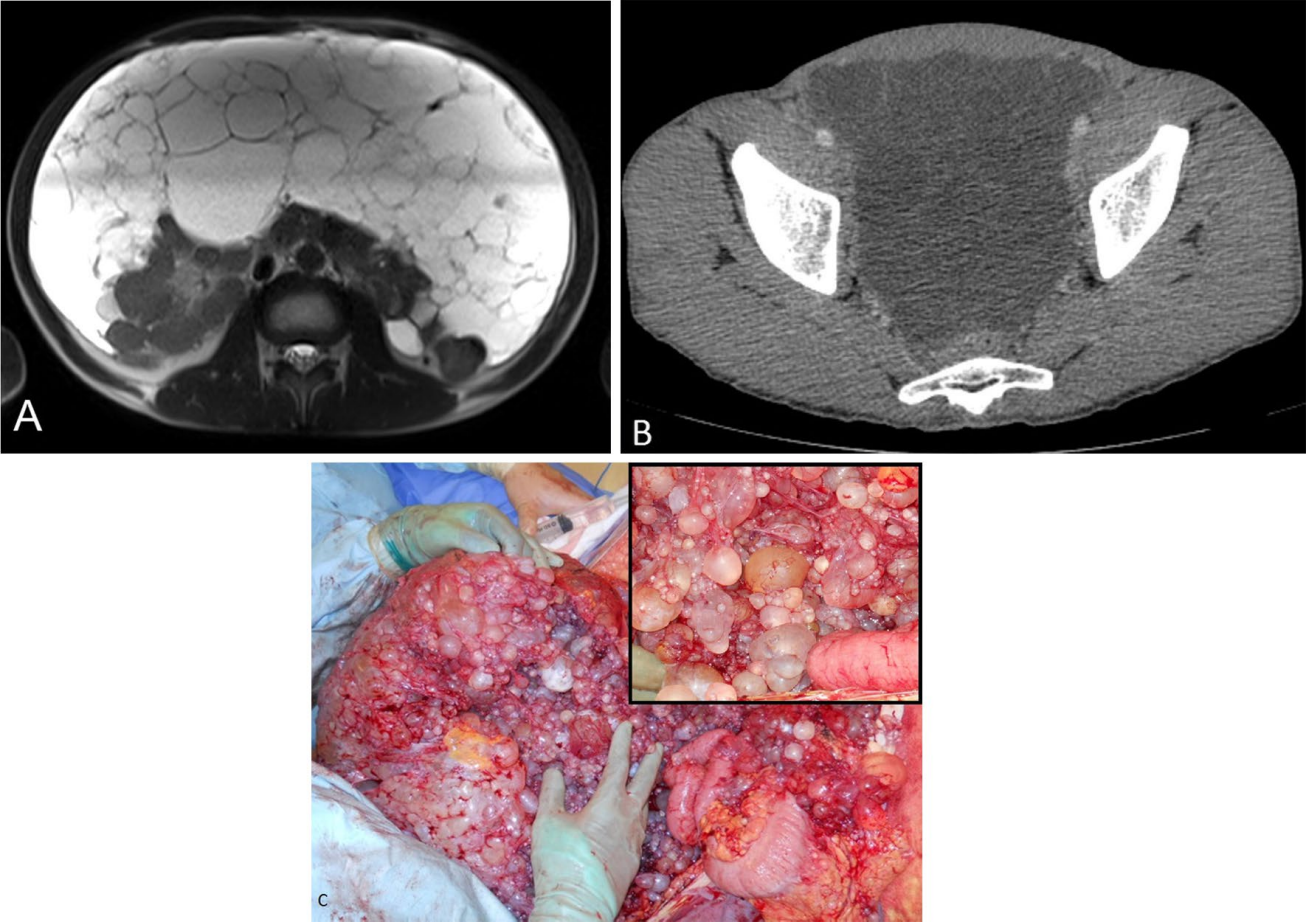
**a**–**c** 42-year-old male with diffuse form of cystic mesothelioma. **a** MRI T2 weighted axial image shows diffuse multi-cystic lesions in the peritoneal cavity. **b** CT through the pelvis shows fluid attenuation cystic lesions with fine non-enhancing septations. **c** Intra-operative photograph shows diffuse multi-loculated cystic masses in the diffusely filling the peritoneal cavity

**Fig. 3:**
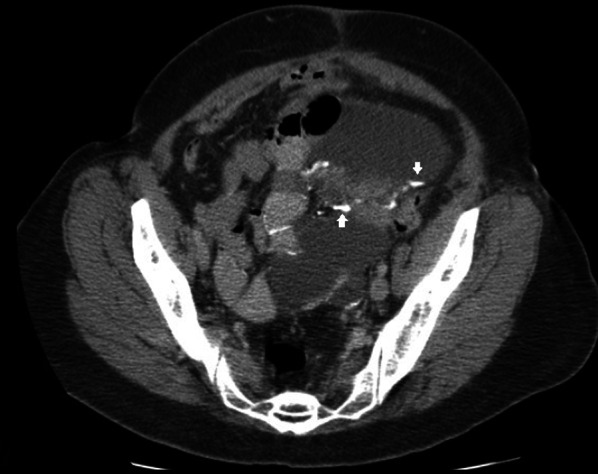
CT axial image of a 47-year-old male patient with cystic mesothelioma showing nodular calcifications along the cyst walls (arrows)

CT and MRI are the modalities of choice for imaging cystic mesothelioma. The radiological appearances are non-enhancing, thin-walled multi-loculated cystic lesions, usually centred in the pelvis and para-colic gutters and typically the cysts surround the pelvic organs (Fig. 1) [21–23]. In some cases, cystic lesions may be seen diffusely in the peritoneal cavity with more than half showing lesions in the small bowel mesentery and the greater omentum (Fig. 2) [21]. Calcification of the cyst walls (Fig. 3) has been rarely reported [24]. The differential diagnoses for cystic mesothelioma include lymphangioma, mesenteric and omental cysts, cystic ovarian masses, pseudomyxoma peritonei, endometrioma and peritoneal hydatidosis. As a rare entity, pre-operative diagnosis of cystic mesothelioma is seldom made except in specialised centres and histological evaluation is required to confirm the diagnosis.

## Well-differentiated papillary peritoneal mesothelioma

**Fig. 4:**
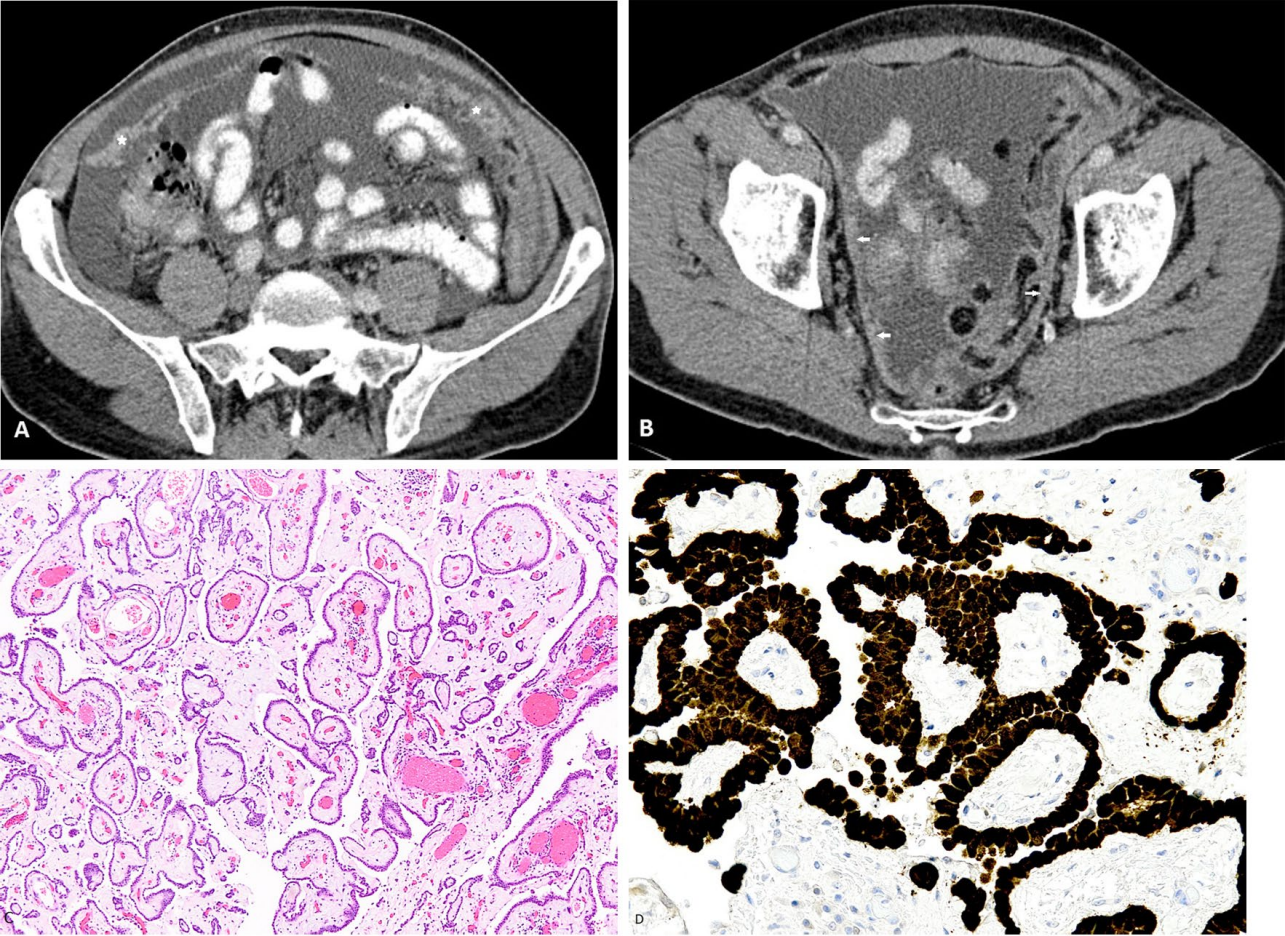
**a**–**d** 42-year-old female with well-differentiated papillary mesothelioma of the peritoneum. **a**, **b** CT axial images showing moderate volume ascites, plaque-like peritoneal thickening (arrow) and omental cake (*). **c** Low power photomicrograph (H&E, × 4) shows fibrovascular cores covered by a layer of cuboidal mesothelial cells. **d** High-power photomicrograph (calretinin immunostaining, × 20) showing expression of calretinin by the cells confirming mesothelial origin

Well-differentiated papillary mesothelioma is uncommon and is seen mainly in females. This subtype has no clear association with asbestos, although anecdotally some patients give a history of asbestos exposure, and it has an indolent clinical course and a favourable prognosis [25, 26]. More than half of the patients with this variant have peritoneal-based masses less than 1 cm in size, which are mostly detected incidentally, either during surgery or on imaging performed for other unrelated causes [27]. Imaging findings range from large volume ascites, omental caking and nodular or plaque-like peritoneal thickening (Fig. 4). Nodular calcifications can be seen in this subtype [25, 28].

## Malignant peritoneal mesothelioma

**Fig. 5:**
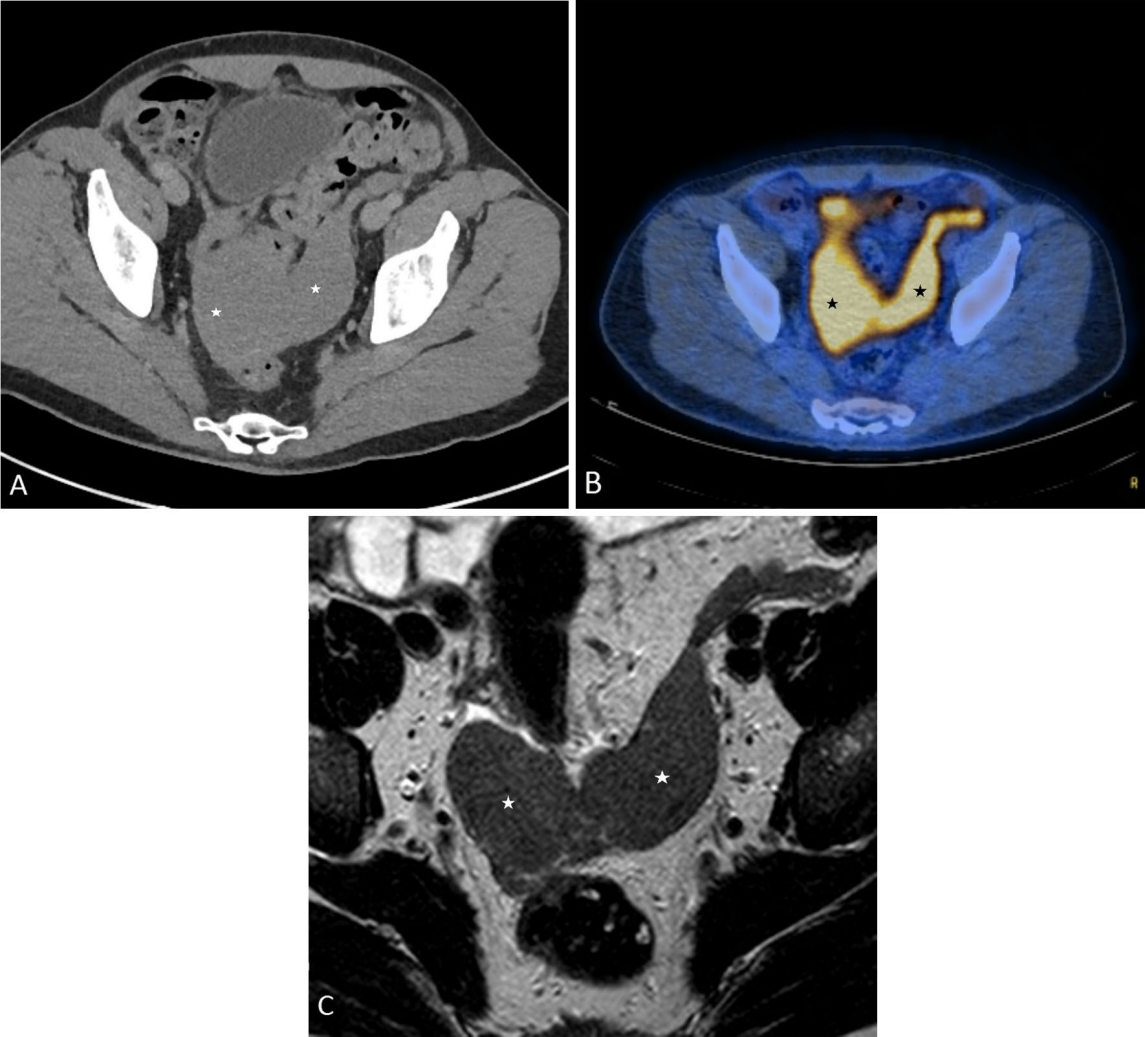
**a**–**c** 48-year-old male with ‘dry’ appearance of epithelioid type of malignant peritoneal mesothelioma. **a**, **b** Axial PET-CT images through the pelvis showing omental caking (*), nodular mesenteric fold thickening (arrows). **b** Axial image through the pelvis showing soft tissue density, FDG-avid, plaque-like, nodular soft tissue thickening of the pelvic peritoneum (*). **c** MRI T2 axial image showing T2 intermediate signal intensity, nodular soft tissue (*) along the pelvic peritoneum

**Fig. 6:**
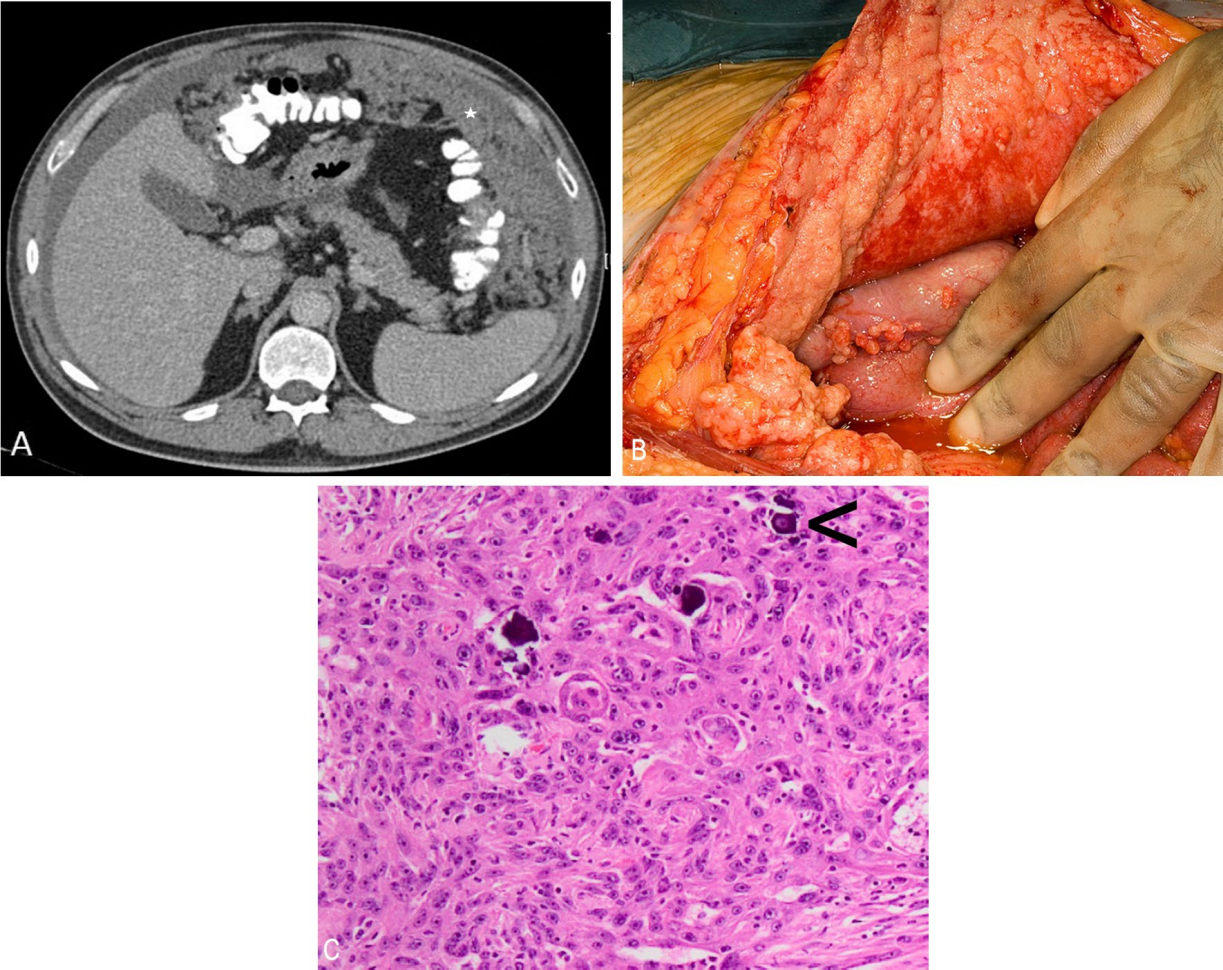
**a**–**c** 56-year-old male with ‘wet’ appearance of epithelioid peritoneal mesothelioma. **a** CT axial images showing omental cake (*) and ascites **b** Intraoperative photograph showing omental cake, peritoneal thickening and ascites. **c** High-power photomicrograph (H&E, × 20) demonstrates pleomorphic sheets of epithelioid cells with large nuclei, prominent nucleoli and eosinophilic cytoplasm. A few psammoma bodies are visible; one is marked with an arrowhead

Malignant peritoneal mesothelioma is more common among older men, in their fifth and sixth decades and has a strong association with asbestos exposure. Epithelioid, sarcomatoid and mixed or biphasic mesothelioma are the malignant histological subtypes of peritoneal mesothelioma. Malignant peritoneal mesothelioma tends to spread in a sheet-like fashion along the visceral and the parietal peritoneal surfaces and subsequently becomes confluent, encasing the abdominal viscera. Imaging findings reflect this nature of spread. Based on the predominant imaging findings, malignant peritoneal mesothelioma has been classified as a ‘dry’ painful type, which presents with peritoneal masses (Fig. 5), and a ‘wet’ type, which presents with ascites, omental and peritoneal thickening (Fig. 6) [29].

**Fig. 7:**
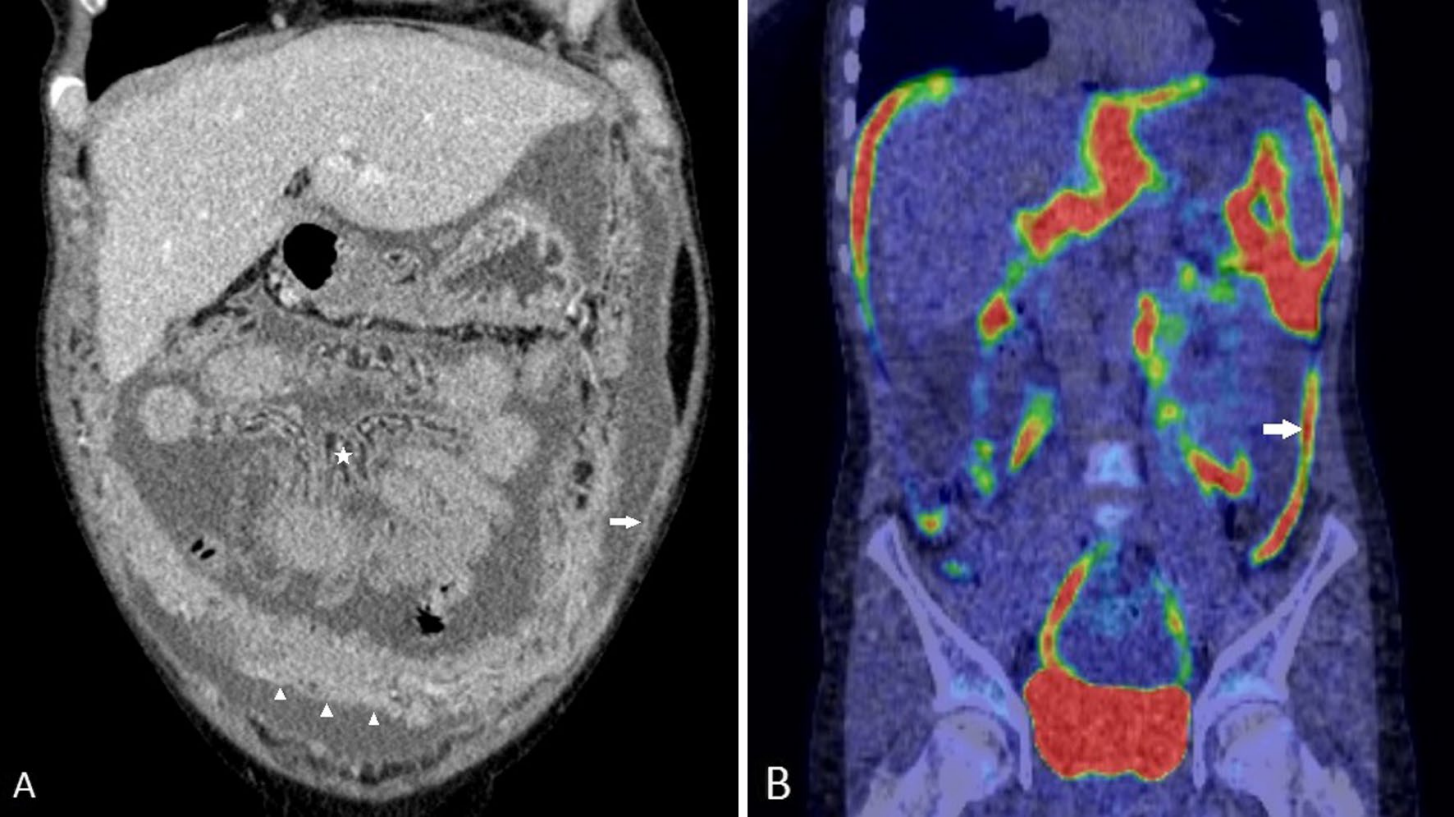
**a**, **b** 47-year-old female with epithelioid type of malignant peritoneal mesothelioma. **a** Coronal CT image demonstrates diffuse plaque-like peritoneal thickening (white arrow), omental caking (arrow heads), ascites and tethered mesentery (*). **b** Coronal PET-CT shows FDG-avid plaque-like thickening (arrow) of the peritoneum

Epithelioid mesothelioma is the commonest subtype of peritoneal mesothelioma [30]. This is a steadily progressive, malignant type with varied imaging findings depending on the severity of disease at the time of diagnosis. Typical imaging findings of epithelioid peritoneal mesothelioma constitute ascites, omental caking, diffuse plaque-like parietal and visceral peritoneal thickening, mesenteric nodules and mesenteric fold thickening (Fig. 7). With increase in severity of malignant peritoneal mesothelioma, there is progressive thickening of the visceral and parietal peritoneum, which eventually encases the solid intra-abdominal organs and the bowel, giving rise to a rind of soft tissue surrounding these structures (Fig. 15).

**Fig. 8:**
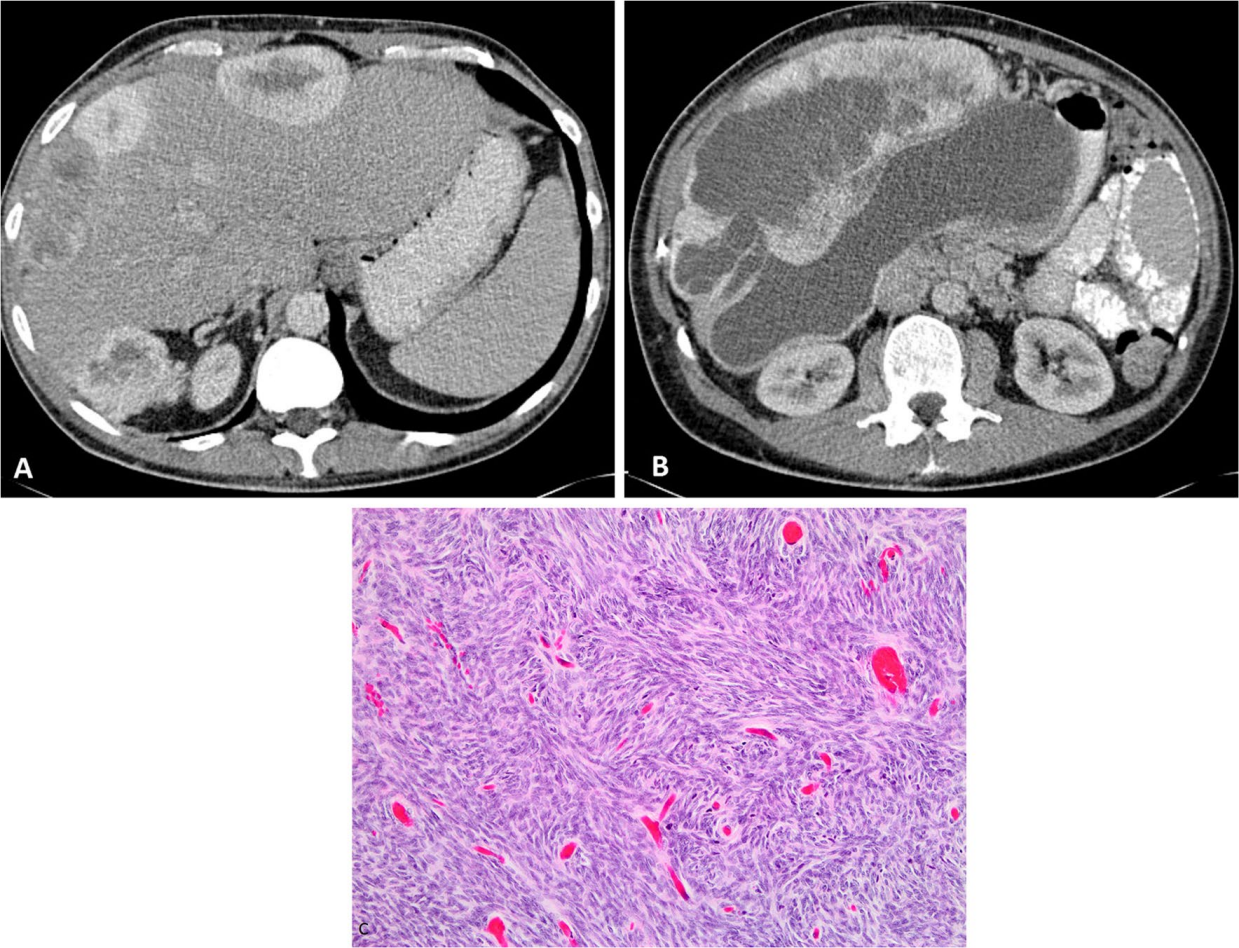
**a**–**c** 57-year-old male with sarcomatoid peritoneal mesothelioma. **a**, **b** CT showing multiple intensely enhancing perihepatic peritoneal-based solid masses with central necrosis. **c** High-power photomicrograph (H&E × 20) showing spindle shaped tumour cells seen in sarcomatoid mesothelioma

**Fig. 9:**
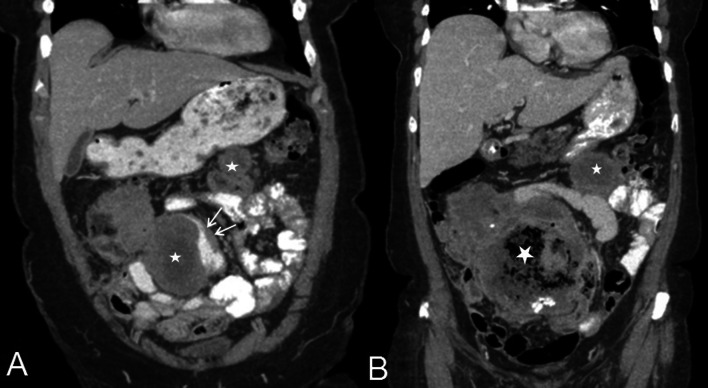
**a**, **b** 78-year-old male with biphasic peritoneal mesothelioma. Serial CT scans show rapidly progressive disease. **a** Coronal CT showing large soft tissue mass in the mesentery (*) indenting and distorting the small bowel and causing eccentric bowel wall thickening (arrows). **b** Coronal CT section through the same site three months later shows increase in the size of the mass and air pockets within suggestive of contained bowel fistulation and disease progression

Sarcomatoid mesothelioma is the most aggressive form of peritoneal mesothelioma with a rapidly progressive clinical course. This subtype is more common in elderly patients in their sixth and seventh decades with a slight male predominance. It is rare in the peritoneal cavity. Sarcomatoid peritoneal mesothelioma presents with large peritoneal-based masses, which can be either localised (Fig. 8) or diffuse [21]. Mixed or biphasic mesothelioma has features of both epithelioid and sarcomatoid subtypes (Fig. 9).

## Treatment of peritoneal mesothelioma

Cystic mesothelioma usually has a slowly progressive clinical course with some reports that it can rarely transform into malignant mesothelioma [31]. The treatment of choice is total surgical excision. However, following total excision the recurrence rates are high, in the range of 45–50% [18, 32]. Due to the local recurrence rates and the rare possibility of malignant transformation, cystic mesothelioma has been rightly reclassified as a borderline malignant neoplasm. For this reason, many centres have adopted an aggressive therapeutic approach, combining CRS with HIPEC to treat cystic mesothelioma with a reported reduction in recurrence rates to 16.7% and significantly improved 5- and 10-year disease-free survival rates for these patients [4, 33].

Treatment of malignant peritoneal mesothelioma is challenging. The diffuse nature of disease means that it is not amenable to radiotherapy as treatment. Following chemotherapy with platinum-based agents, like cisplatin and gemcitabine as used for pleural mesothelioma, the median survival rate is between 6 and 9 months [34]. Median survival rates have improved to 12.1–26.8 months when platinum agents are combined with pemetrexed, but toxicity is high and response rates are poor [34–36]. For these reasons, loco-regional treatment of the peritoneal cavity with cytoreductive surgery and HIPEC has been proposed and utilised as a treatment for peritoneal mesothelioma. Studies in specialised centres have reported superior survival benefits of CRS and HIPEC, when compared with systemic chemotherapy, with an overall median survival of 53 months and 1-, 3- and 5-year survival rates after CRS and HIPEC of 81%, 60% and 47%, respectively [35, 37].

**Fig. 10:**
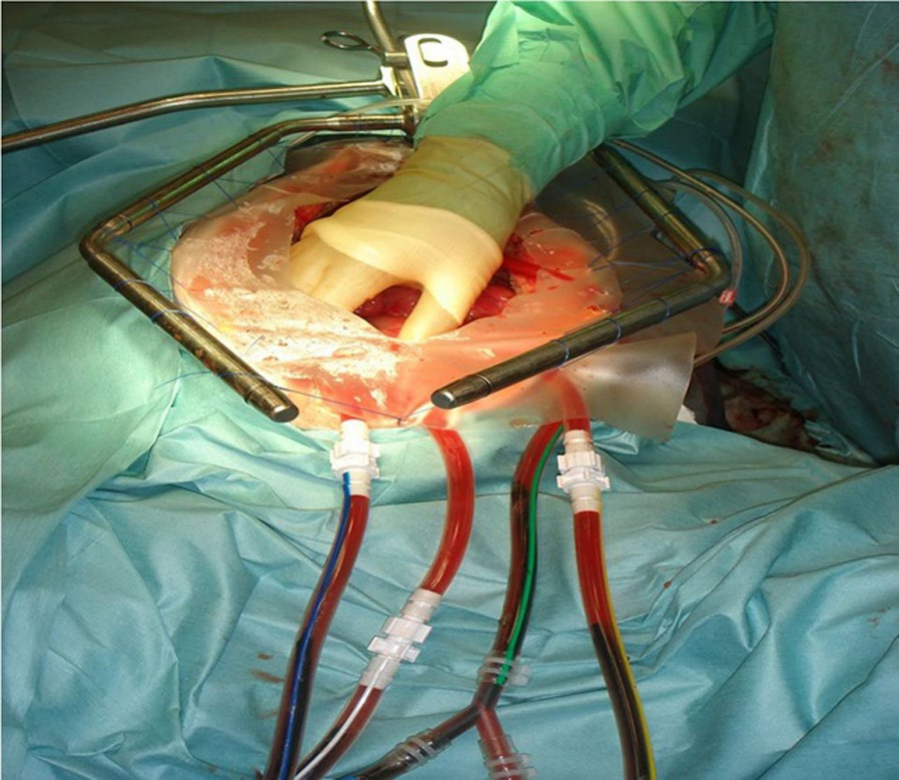
Photograph of hyperthermic intraperitoneal chemotherapy (HIPEC) procedure

The aim of cytoreductive surgery is to remove all visible tumour from the abdominal cavity, or, in other words, to achieve complete cytoreduction. This is achieved by stripping the involved peritoneum (left and right parietal, left and right diaphragmatic and pelvic), performing a liver capsulectomy, an omentectomy and by removing any visible tumour nodules from the mesentery. Involved non-vital viscera are also removed and may include the colon, rectum, ovaries and uterus, gall bladder, spleen or the stomach. Cytoreduction is followed by HIPEC, during which heated chemotherapy is infused into the abdominal cavity for 60–90 min, either by an open (Fig. 10) or a closed technique. Cytoreductive surgery is a major undertaking with an operative mortality of 0.6–4.4% and a morbidity of 7–49%, even in high volume centres [38]. Surgery lasts around 9 h on an average and patients need multidisciplinary support including intensive care. Many patients will require a temporary or permanent stoma. Achieving complete cytoreduction is key to successful outcome [11, 37–43]. Incomplete cytoreduction is associated with poor outcomes and negates the benefits of such a high-risk procedure in most cases. Non-invasive imaging plays an important role in the pre-operative assessment of these patients by identifying disease extent, involvement of key anatomic structures which affect operability and the likelihood of achieving complete cytoreduction.

## Role of imaging in the surgical decision-making

The main role of imaging in assessing patients with peritoneal mesothelioma is to estimate the overall disease burden. Optimal radiological staging helps in assessing patients’ suitability for surgery and prognosis. Methodical assessment for key anatomical structures on imaging is helpful for selecting patients who may potentially benefit from cytoreductive surgery and HIPEC and estimating the extent of the surgery needed. The acronym ‘PAUSE’ has been proposed to summarise the key imaging findings which radiologists must report in a patient with peritoneal malignancy [44]. In this review, we use this concept to elaborate the findings which impact surgical decision-making in patients with peritoneal mesothelioma. The term 'PAUSE' incorporates the following: P, primary tumour and peritoneal carcinomatosis index (PCI); A, ascites and abdominal wall involvement; U, unfavourable sites of involvement; S, small bowel and mesenteric disease; E, extra peritoneal metastases.

### P—peritoneal cancer index (PCI)

**Fig. 11:**
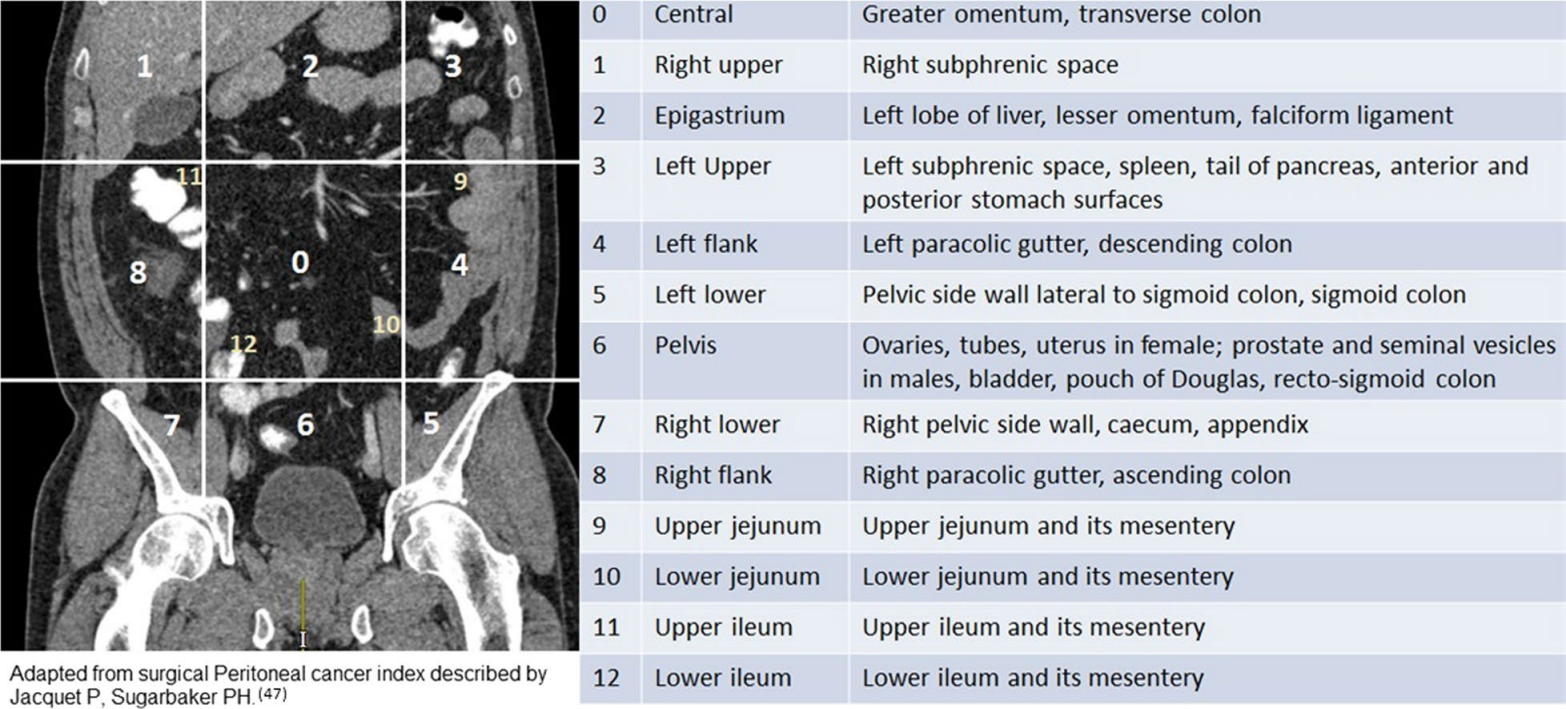
Radiological peritoneal cancer index (rPCI) adapted from surgical peritoneal cancer index (PCI) described by Jacquet et al. [47]. This figure is being reused from author’s prior work published in clinical radiology [44] with permission from Elsevier

The peritoneal cancer index (PCI) was designed, and validated, as a method of estimating the tumour burden during laparotomy and thus was not initially used for case selection [45]. However, PCI closely relates to prognosis of virtually all peritoneal malignancies and correlates with the success of CRS and HIPEC [29, 30, 43]. PCI is a sum of scores obtained from the longest measurement of the largest peritoneal-based lesion in the thirteen anatomical sites of the peritoneal cavity (Fig. 11). The lesions are given a score of 1–3 based on size: 1 if < 0.5 cm, 2 if 0.5–5 cm and 3 if > 5 cm. Imaging is now widely used to pre-operatively estimate the radiological PCI and thus help in patient selection for CRS and HIPEC.

The PCI cut-off for survival benefits largely depends on the histological subtype of peritoneal mesothelioma. For example, PCI estimation is less relevant in determining the outcome of cystic mesothelioma which has a favourable outcome despite its extent. On the other hand, the outcome of malignant peritoneal mesothelioma closely relates to the PCI. Based on a multicentre study, a novel TNM staging was proposed for patients with malignant peritoneal mesothelioma. In this method, the authors propose T-staging of peritoneal mesothelioma based on PCI with PCI of 1–10, 11–20, 21–30 and 30–39 being T1 to T4 stages, respectively [46]. Based on this staging, the 5-year survival of patients with stage I (T1, N0, M0), II (T2/T3, N0, M0) and III (T4, N1, M1 or any T, N1, M1) malignant peritoneal mesothelioma was 87%, 53% and 29%, respectively. This illustrates the importance of PCI in patients with peritoneal mesothelioma and provides a rationale for pre-operative assessment of radiological PCI.

CT is the most widely used imaging modality for estimating radiological PCI. However, CT underestimates PCI and has been reported by some to be only half of that found at surgery [47]. Moreover, CT has poor sensitivity for detecting small peritoneal nodules less than 0.5 cm [48]. Despite these limitations, CT remains the modality of choice for the initial evaluation of patients with peritoneal mesothelioma since it is often the index investigation. A radiological study of 59 patients with peritoneal mesothelioma showed significantly higher rPCI (34 vs. 17), large volume upper abdominal disease, small bowel and mesenteric disease among patients who had incomplete cytoreduction for malignant peritoneal mesothelioma compared to those who had complete cytoreduction [21]. CT or MRI-derived rPCI of ≥ 30 was associated with worse survival and rPCI of ≤ 19 was associated with improved survival in another study of 53 patients with malignant peritoneal mesothelioma [49]. Thus, CT is a good technique for identifying patients with high PCI disease, who may not benefit from CRS and HIPEC.

The CT protocol should include intra-venous contrast enhanced CT of the chest, abdomen and pelvis with thin Sections (1–2 mm) CT of the abdomen and pelvis in the portal venous phase. We advocate positive oral contrast given 1.5 h prior to the study in order to opacify the small bowel. High-quality focussed MRI may be superior to CT in detecting peritoneal disease and can estimate PCI with 88–91% accuracy [47, 48]. However, long scan time, patient-related contra-indications and capacity issues limit the wider usage of MRI in many patients. After an initial assessment with CECT, MRI is usually done in select patients who might be candidates for surgery. MRI is performed with the view to detecting disease which may be missed on CT. But in patients who undergo laparoscopic biopsy, the surgeon would use this opportunity to assess the disease burden and MRI will not add to the management in these patients unless adhesions prelude optimal laparoscopic inspection of the peritoneal cavity. The role of PET-CT in estimating PCI is unclear but in our experience is of limited value [50, 51].

### A—abdominal wall involvement and ascites

**Fig. 12:**
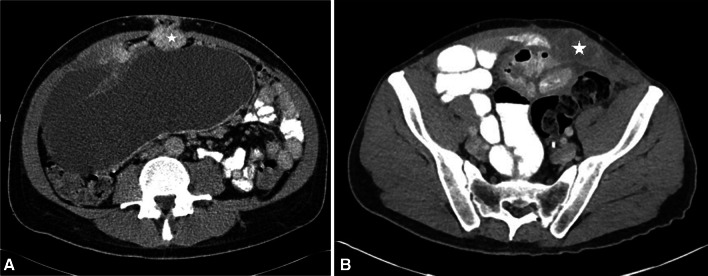
**a**, **b**: Abdominal wall disease in two different patients with malignant peritoneal mesothelioma. **a** CT of a patient with sarcomatoid type of peritoneal mesothelioma showing enhancing soft tissue nodule (*) in the abdominal wall. **b** CT of a patient with epithelioid mesothelioma shows a large lateral abdominal wall mass (*) abutting and tethering small bowel loop

Spread of peritoneal mesothelioma to the abdominal wall is not a definite contraindication for surgery. However, this may negatively impact on the surgery required and outcome after surgery. Port-sites; drain sites; surgical wounds and scars are common locations of abdominal wall disease [52]. While midline (Fig. 12a) abdominal wall lesions can be easily excised, lateral (Fig. 12b) abdominal wall lesions are problematic and often incurable due to the nature of the abdominal wall lympho-vascular supply. Also, adhesions between the bowel loops and the abdominal wall lesions can compromise the view during diagnostic laparoscopy precluding optimal assessment of disease and increasing the risk of entero-cutaneous fistula. Overall, abdominal wall lesions increase the morbidity of cytoreductive surgery and complex reconstructive techniques may be needed to close the abdomen [53].

Unlike the malignant ascites from peritoneal carcinomatosis, ascites from peritoneal mesothelioma does not directly impact on the surgery or the outcome. However, complications of ascites such as weight loss, protein malnutrition, renal failure and spontaneous bacterial peritonitis can adversely affect outcome [54].

### U—unfavourable sites

**Fig. 13:**
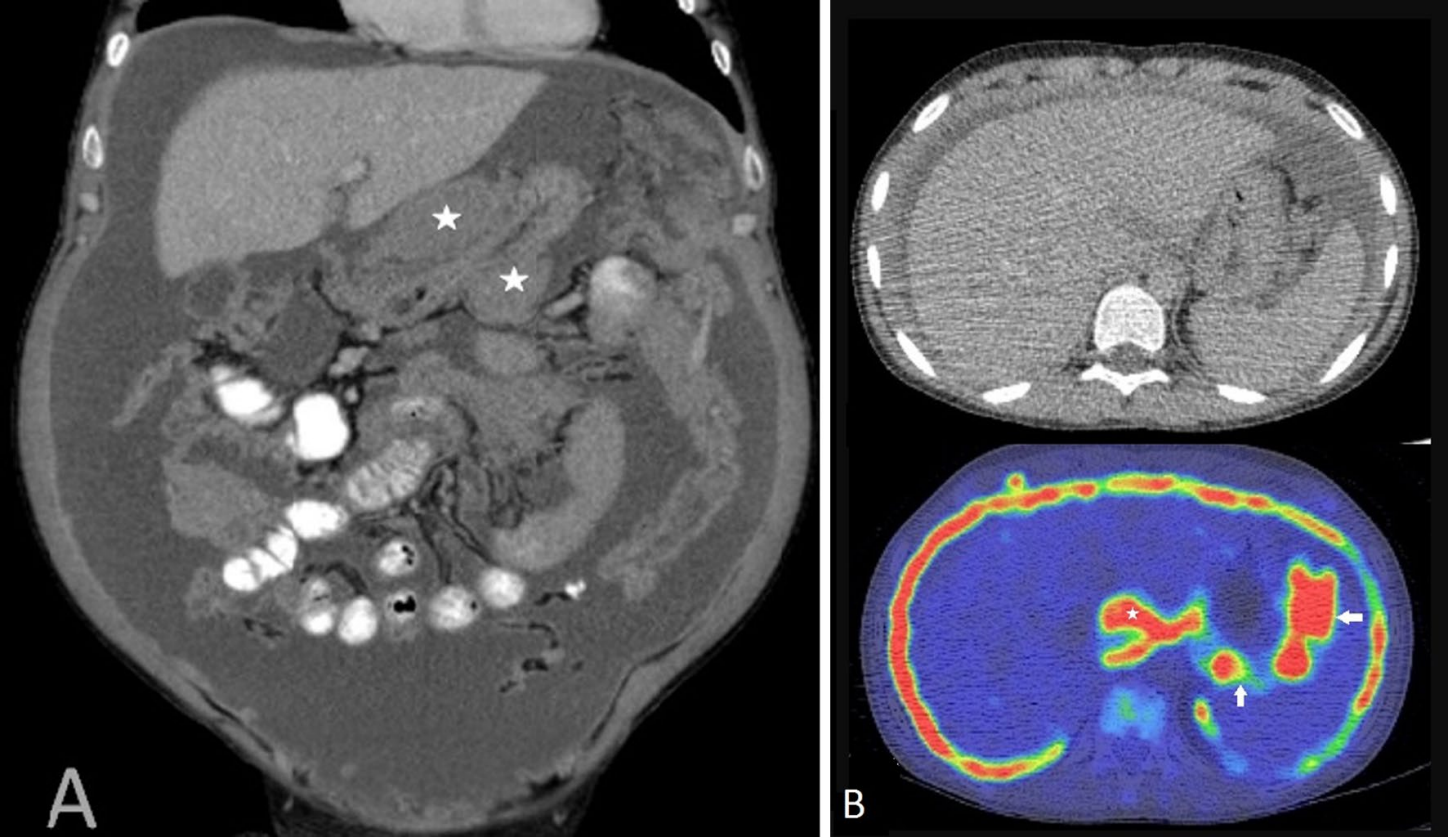
**a**, **b** Images of two patients with epithelioid malignant peritoneal mesothelioma showing disease in the epigastric region. **a** Coronal CT with extensive peri-gastric disease (*) seen as soft tissue masses around the stomach. Also note the tethered mesentery. **b** Axial PET-CT images showing FDG-avid disease in the lesser omentum, lesser sac, porta (*) and peri-gastric region (arrows)

**Fig. 14:**
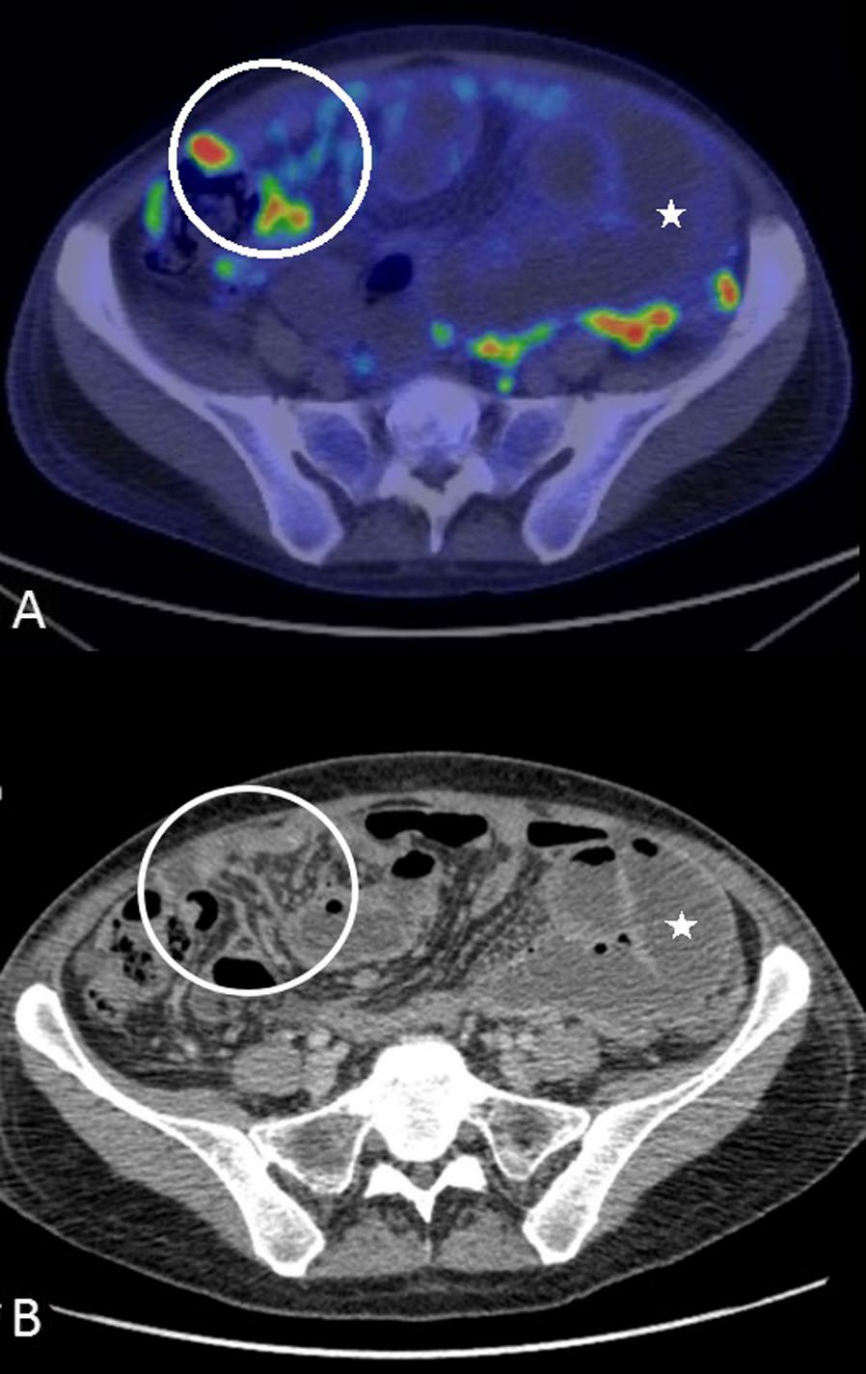
**a**, **b**: **a** PET-CT and (**b**) CT images of a patient with epithelioid malignant peritoneal mesothelioma showing nodular mesenteric fold thickening (white circle) and segmental small bowel obstruction(*)

**Fig. 15:**
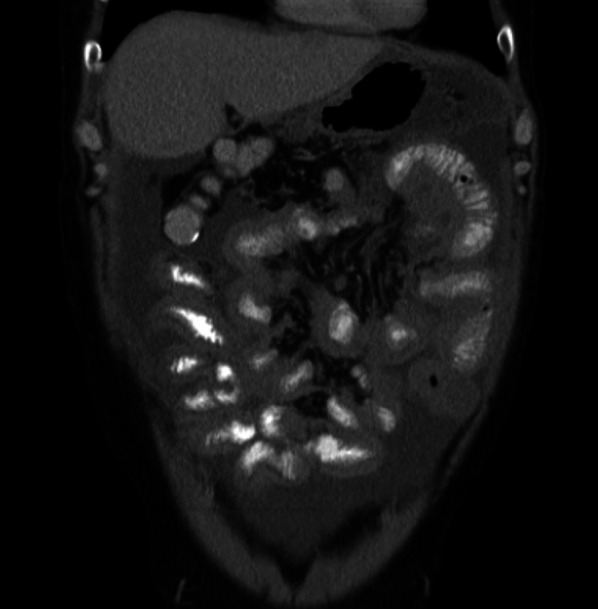
CT scan, coronal image of a patient with advanced epithelioid malignant peritoneal mesothelioma showing a rind of soft tissue around the small bowel due to diffuse thickening of the small bowel serosa or the visceral peritoneum

**Fig. 16:**
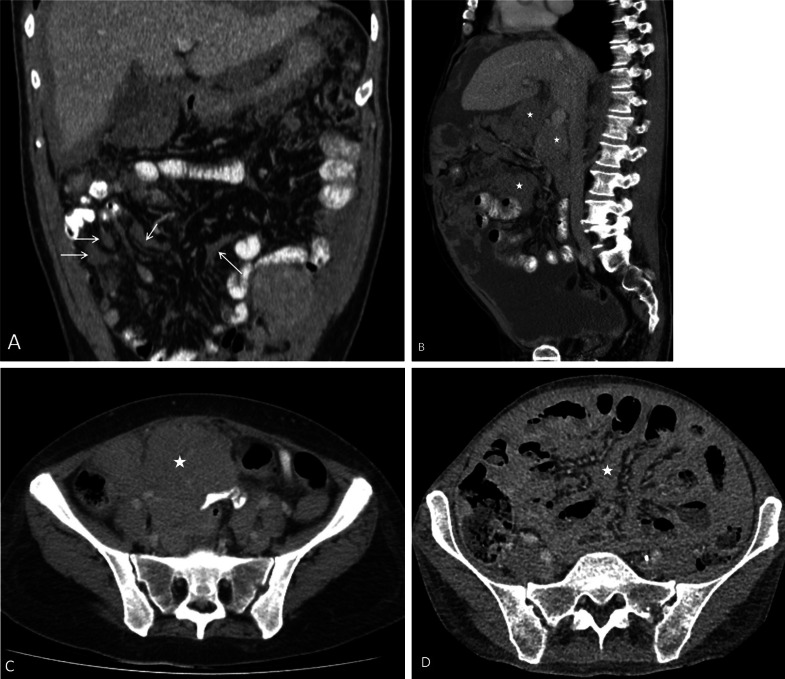
**a**–**d** CT images of different patients with malignant peritoneal mesothelioma showing findings of advanced mesenteric disease. **a** Nodular mesenteric fold thickening (arrows). **b** Sagittal CT showing soft tissue masses in the root of mesentery (*). **c** Mesenteric soft tissue mass (*) encasing small bowel. **d** Stellate mesentery on CT, which represents gross nodular thickening and retraction of the mesentery (*)

**Fig. 17:**
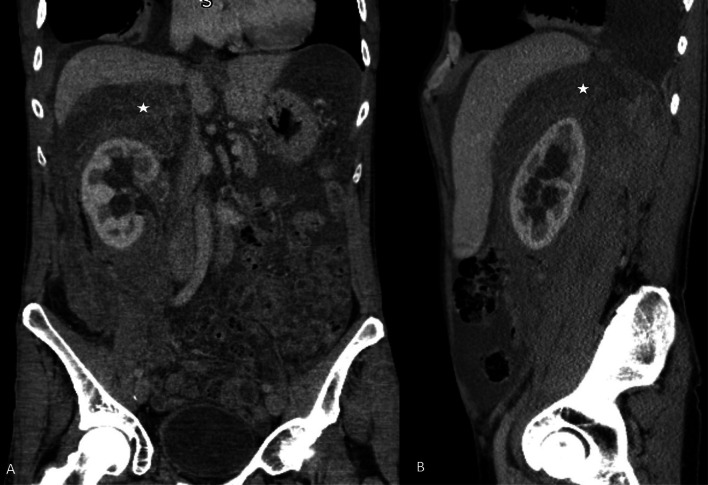
67-year-old male with biphasic type of malignant peritoneal mesothelioma. **a**, **b** CT coronal and sagittal reformatted images showing right perinephric soft tissue density mass (*), right hydronephrosis and ascites. This patient also had concurrent right intra-thoracic disease

**Fig. 18:**
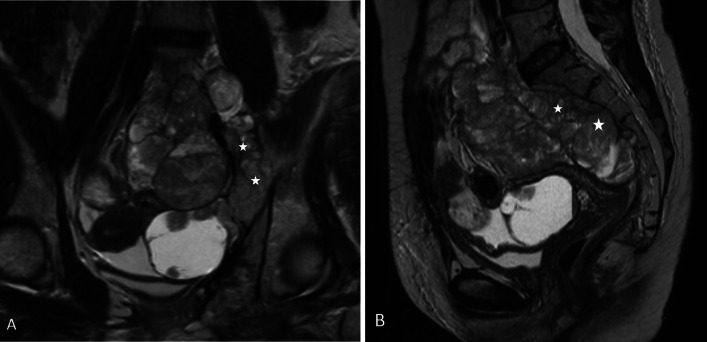
**a**, **b** MRI images of a patient with biphasic malignant peritoneal mesothelioma. **a** Coronal MRI of the pelvis shows a large mixed solid cystic mass in the pelvis involving the left pelvic side wall (*). **b** Sagittal MRI of the pelvis shows the mass extending into presacral space (*). These constitute U2 findings which render complete cytoreduction unlikely

Irrespective of the peritoneal cancer index (PCI), involvement of certain anatomic structures by disease negatively impacts on surgery and outcome. We have labelled disease in these key anatomic sites as ‘unfavourable’. For ease of communication, we have further scored these sites as U0, U1 and U2 sites based on impact on management (Table 1) [44]. U0 indicates that no unfavourable sites are detected on imaging and thus complete cytoreduction may be feasible. Involvement of U1 category sites on imaging suggests that cytoreductive surgery may still be possible; however, a more complex surgical procedure should be anticipated. Disease at U2 sites suggests that complete cytoreduction is unlikely to be achieved. Figures 13, 14, 15, 16, 17 and 18 show examples of malignant peritoneal mesothelioma demonstrating disease in one or more unfavourable sites. Careful radiological review aimed at identifying disease in unfavourable sites during MDT discussions is critical for choosing patients who would benefit from cytoreductive surgery and for planning the surgery [55].

**Table 1:** Review areas for unfavourable sites [44] (published with permission from clinical radiology)

U1* sites	U2^**^ sites
*Disease in the epigastric region*	Biliary obstruction due to tumour
Lesser omentum/lesser sac/around the left lobe of liver/fissures/falciform ligament/ligamentum teres/encasement of left gastric artery	
Porta hepatis/porto-caval space/gallbladder fossa/near hepatic vein or IVC	Coeliac, periportal, epiphrenic and retroperitoneal nodes
Spleen	
Stomach encasement	Root of mesentery/ligament of treitz
Peri-pancreatic and para-duodenal space	
*Disease in the retroperitoneum*	DJ flexure, most of the proximal small bowel involved, small bowel obstruction, stellate mesentery
Hydronephrosis and ureteric involvement	
Psoas, iliacus and quadratus lumborum muscles	
*Pelvis*	Pelvic side wall disease/nodes
Bladder trigone	
Seminal vesicle, prostate	
Disease encasing the external iliac vessels	Disease involving the sacrum

*U1 sites increase surgical complexity and may need, for example, gastrectomy, Whipple’s procedure, nephrectomy, ureteric reimplantation, cystectomy or prostatectomy

**U2 sites reduce the likelihood of complete cytoreduction

### S—small bowel and mesentery

**Fig. 19:**
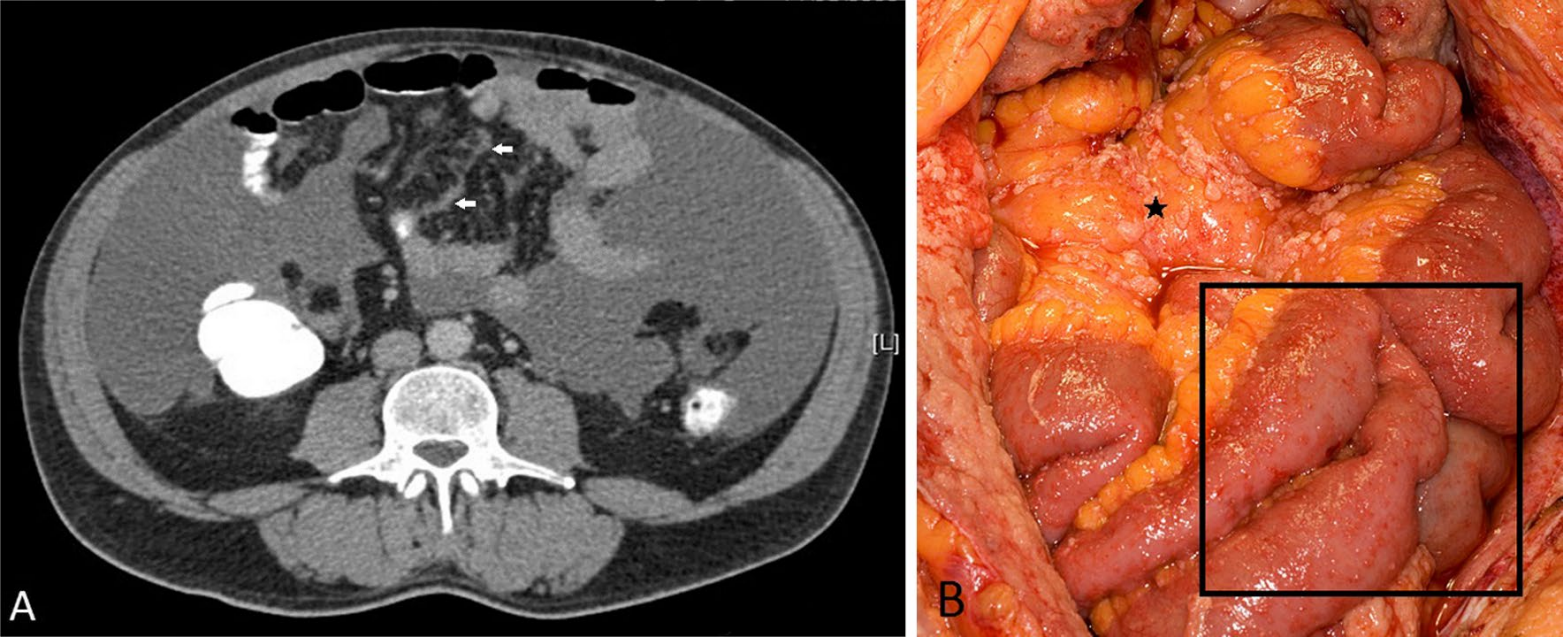
**a**, **b** Patient with epithelioid peritoneal mesothelioma. **a** CT shows ascites and subtle mesenteric nodularity (arrows). **b** Photograph from diagnostic laparoscopy showing diffuse mesenteric nodules (asterisk) and diffuse small bowel serosal nodules (black rectangle)

The limiting factor in achieving complete cytoreduction is commonly extensive involvement of the small bowel and mesentery. While focal or regional disease in the small bowel and mesentery may be amenable to complete cytoreduction, this is unlikely when there is multi-focal or diffuse small bowel and mesenteric disease [21, 55, 56]. CT is the most common initial imaging modality used for assessing patients with peritoneal mesothelioma. However, it has been reported that CT has poor overall accuracy of only 21–25% for detecting small bowel and mesenteric disease compared to 92% for MRI [47, 48]. This is mainly because diffuse plaque-like and miliary disease is more often missed on CT (Fig. 19) and CT has poor sensitivity for nodules smaller than 0.5 cm [48]. Thus, in patients being considered for cytoreductive surgery of curative intent, most centres resort to pre-operative diagnostic laparoscopy to complement CT and some perform MRI in patients with inconclusive CT, in order to exclude subtle diffuse small bowel and mesenteric disease.

During CT examination, the use of oral contrast, either neutral or positive, is useful for delineating disease in the small bowel or mesentery. In our experience, the use of positive oral contrast is most useful in identifying small bowel and mesenteric disease, especially in a setting of diffuse peritoneal disease with predominantly isodense or hypodense peritoneal disease. When large soft tissue masses are the predominant finding, the use of neutral contrast medium may be sufficient to allow appreciation of bowel wall enhancement and thickening.

The earliest findings of small bowel and mesenteric disease, detected on CT, are kinking of the small bowel loops, distortion of the bowel lumen and subtle bowel wall thickening. With extensive disease, there are soft tissue masses in the mesentery; mesenteric fold thickening and nodularity; tethering of the mesentery which progresses to stellate mesenteric retraction; eccentric/concentric bowel wall thickening; a rind of soft tissue around the bowel due to diffuse visceral peritoneal thickening and segmental small bowel obstruction (Figs. 14, 15 and 16) [57]. In patients with findings pointing to extensive small bowel and mesenteric disease, complete cytoreduction is unlikely to be achieved and these findings constitute U2 disease [44]. In a study describing the utility of CT for selecting patients for cytoreductive surgery in patients with peritoneal mesothelioma, Yan et al. classified degree of small bowel and mesenteric disease as class I, II and III based on similar imaging findings [56]. Class 1 disease had only ascites; bowel wall thickening and mesenteric soft tissue masses were categorised as class II disease; and complete loss of mesenteric architecture and bowel obstruction constituted class III findings [56].

In contrast to malignant peritoneal mesothelioma, in patients with cystic mesothelioma, the multi-loculated cystic lesions seen in the omentum, the small bowel and the mesentery are usually free lying lesions which are amenable for surgical excision. Thus, CT findings suggestive of small bowel and mesenteric involvement should only be applied to patients with malignant peritoneal mesothelioma [21].

### E—extraperitoneal disease

**Fig. 20:**
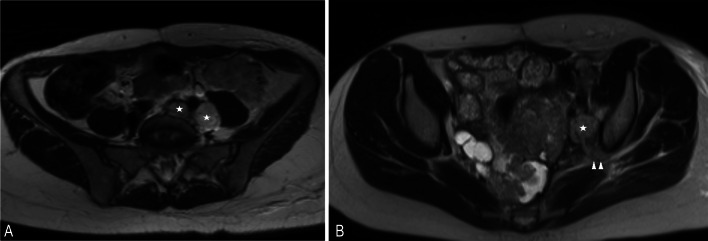
**a**, **b** Two different patients with biphasic malignant peritoneal mesothelioma. MRI shows (**a**) retroperitoneal (*) nodes and (**b**) left pelvic side wall lymph node (*). Note the disease extension into the left sciatic notch (**b**, arrow heads)

**Fig. 21:**
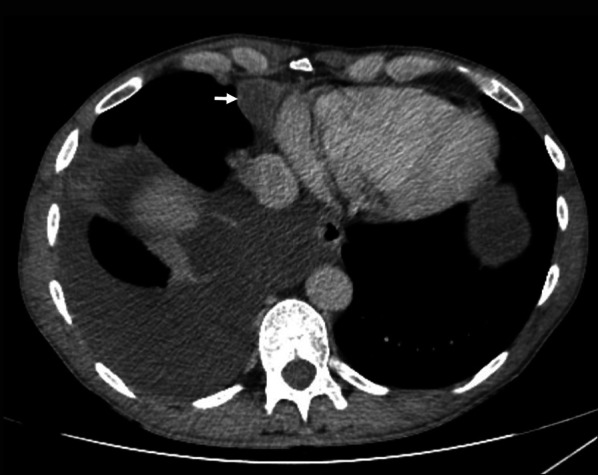
CT image of a patient with malignant peritoneal mesothelioma and concurrent pleural disease shows pleural based nodules (arrow) and pleural effusion

Extra-peritoneal spread of peritoneal malignancy is suggestive of systemic spread and advanced stage. Malignant peritoneal mesothelioma is known to metastasise to the lymph nodes (Fig. 20), pleural cavity (Fig. 21), pericardium, lungs and liver and these are considered as significant extra-peritoneal disease [3]. PET-CT has an important role in helping to exclude significant extra-peritoneal disease in patients who are being considered for cytoreductive surgery [50, 58].

## Structured reporting of peritoneal malignancy

In a large multi-centric UK-wide peritoneal malignancy MDT, 155 patients with peritoneal mesothelioma were reviewed between 2016 and 2018. In this series, imaging played an important role in decision-making at the MDT and presence of unfavourable disease sites and small bowel or mesenteric disease preluded surgery. Twenty-two (14.2%) patients underwent CRS and HIPEC and 19 had complete cytoreduction [55]. The median surgical PCI of operated patients was 17, which was similar to the median rPCI found in a previous imaging study among patients who underwent complete cytoreduction [21, 55]. Operated patients had better survival outcomes compared to those treated with systemic chemotherapy. Though correlation between ‘PAUSE’ and the outcome is yet to be determined, structured reporting with a score given to each component of ‘PAUSE’ as in Table 2 can be an effective means of communicating and objectively documenting imaging findings in an MDT setting.

**Table 2:** Simplified scoring system to communicate imaging findings in peritoneal malignancy using the concept of ‘PAUSE’

	PAUSE components	Score
P	rPCI**	0–39
A	Ascites	Present—1
		Absent—0
	Abdominal wall involvement	Present—1
		Absent—0
U	Unfavourable sites: U score	U0—0
		U1—1
		U2—2
S	Yan’s CT [56] grade of small bowel involvement	No small bowel and mesenteric—0
		Class 1—1
		Class 2—2
		Class 3—3
E	Extraperitoneal metastasis	Present—1
		Absent—0

**rPCI cut-off used in practice is variable for different types of peritoneal malignancy based on the tumour biology. Based on local practice, rPCI could be dichotomised for the purposes of clinical usage and research

## Conclusions

Radiology has a crucial role in aiding surgical decision-making. ‘PAUSE’ was designed with a focus on key elements determining feasibility, prognosis and potential benefits from cytoreductive surgery and HIPEC. Using ‘PAUSE’ and several examples of peritoneal mesothelioma, we emphasise key imaging features which should be included in radiology reports on patients with known or suspected peritoneal malignancy.

## Data Availability

Data are included in the manuscript.

## Abbreviations

CM: Cystic mesothelioma
CRS: Cytoreductive surgery
HIPEC: Hyperthermic intraperitoneal chemotherapy
MPM: Malignant peritoneal mesothelioma
MTD: Major tumour debulking
rPCI: Radiological peritoneal cancer index
